# Convention on Vital Statistics

**Published:** 1880-03-20

**Authors:** 


### *CONVENTION ON VITAL STATISTICS.*

The National Board of Health requests, through the *Health Bulletin*, Washington, D. C., that all who are interested in vital statistics, and especially those who are charged with the duties of state or municipal registration, to meet with it in Washington, on the 6th of May next, for the purpose of considering the best methods for the collection and publication of such statistics. This convention will consider more especially mortality statistics, for which it is extremely desirable to secure more uniformity than exists at present in nomenclature, in nosological arrangement, and in the forms of tables or graphic representations intended to show the relations of causes of death to locality, meteorology, sex, age, nativity, occupation, and birth-rate.

